# Spatial geometric and magnetic resonance signal intensity changes with advancing stages of nucleus pulposus degeneration

**DOI:** 10.1186/s12891-017-1838-0

**Published:** 2017-11-21

**Authors:** Shu-Hua Yang, Alejandro A. Espinoza Orías, Chien-Chou Pan, Issei Senoo, Gunnar B. J. Andersson, Howard S. An, Nozomu Inoue

**Affiliations:** 1 Department of Orthopedic Surgery, Rush University Medical Center, 1611 W Harrison St., Ste. 201, Orthopedic Bldg, Chicago, IL 60612 USA; 2 Department of Orthopedics, National Taiwan University College of Medicine and National Taiwan University Hospital, Taipei, Taiwan; 3 Taichung Veterans General Hospital, Orthopedic Surgery, Taichung, Taiwan; 4 Department of Orthopedic Surgery, Asahikawa Medical University, Hokkaido Prefecture, Asahikawa, Japan

**Keywords:** Intervertebral disc, Nucleus pulposus, Disc degeneration, Signal intensity, Homogeneity, Geometric center, MRI

## Abstract

**Background:**

With advancing stages of degeneration, denaturation and degradation of proteoglycans in the nucleus pulposus (NP) lead to tissue dehydration and signal intensity loss on T2-weighted MR images. Pfirrmann grading is widely used for grading degeneration of intervertebral discs (IVDs). The criterion to differentiate IVDs of Pfirrmann Grade I from the other grades is NP homogeneity. Pfirrmann grading is qualitative and its assessment may be subjective. Therefore, assessment of quantitative objective measures correlating with early disc degeneration may complement the grading. This study aimed to evaluate the applicability of the distance between the center weighted by signal intensity (weighted center) and the geometric center as a parameter of NP homogeneity. Other phenomena related to advancing stages of degeneration were also investigated.

**Methods:**

MR images of 65 asymptomatic volunteers with a total of 288 lumbar IVDs with clearly identifiable nucleus pulposus boundary (Pfirrmann Grade I, II and III) were included in this study. A custom-written program was developed to determine the IVD longitudinal axis, define the NP boundary, and to locate the coordinates of geometric and weighted NP centers on the mid-sagittal image of each studied IVD. The distances between the weighted and geometric centers on the longitudinal axis and the perpendicular axis of each IVD were calculated.

**Results:**

The weighted center located posterior to the geometric center, which indicated the signal intensity was lower at the anterior portion of the NP, in 85.8% of studied IVDs. The distance between the weighted and geometric center on the longitudinal axis was significantly shorter in homogeneous (Pfirrmann Grade I) than in inhomogeneous (Grade II) IVDs. The distance on the perpendicular axis in Grade III IVDs was significantly larger than that in Grade I and Grade II IVDs.

**Conclusion:**

The relationship between the weighted and geometric centers can serve as an indicator for NP homogeneity. The distance between both centers through advancing stages of degeneration demonstrated decrease of signal intensity progressing along the longitudinal axis initially and then along the cranio-caudal direction at later stages. These findings could provide insights of initiation and subsequent progression of degenerative changes in IVDs.

## Background

Degeneration of the intervertebral disc is a multifactorial disease and is believed to be associated with spinal disorders, including low back pain [1, 2]. The intervertebral disc is comprised of the nucleus pulposus core, the multilaminar annulus fibrosus (AF), and cartilaginous endplates. The nucleus pulposus is rich in proteoglycans and the proteoglycans provide high water content within the nucleus pulposus. During the early stage of intervertebral disc degeneration, loss of proteoglycans and type II collagen has been observed [3]. Magnetic resonance (MR) images can detect dehydration associated with proteoglycan denaturation and degradation as a loss of the signal intensity on T2-weighted MR images [4, 5]. Restoring proteoglycan content is the basis of novel biological strategies for prevention or repair of intervertebral disc degeneration [6].

Pfirrmann grading is widely used to grade intervertebral disc degeneration [7]. This classification system is based on mid-sagittal plane of T2-weighted MR images. In order to distinguish earlier stages of degeneration, observations are focused on the changes in the nucleus pulposus including its homogeneity, signal intensity and distinction as compared with the AF. However, Pfirrmann grade is qualitative and its assessment may be subjective [5, 8]. The most important criterion to differentiate Pfirrmann Grade I disc from the other grades is the nucleus pulposus’ homogeneity. The evaluation of homogeneity has not been defined in a quantified manner yet. Therefore, assessment of quantitative objective measures correlating with early disc degeneration may complement the grading.

By using sagittal images, a center weighted by the signal intensity of T2-weighted MR images within the nucleus pulposus (weighted center) has been introduced as a potential variance for image analysis of the intervertebral disc [9]. The distance between the weighted center and the geometric center of the nucleus pulposus has been used as a predictive factor of the progression of intervertebral disc degeneration. The purpose of this study was to evaluate the applicability of the distance between the weighted center and the geometric center as a parameter of homogeneity for the nucleus pulposus and other phenomena related to advancing stages of degeneration.

## Methods

A total of 65 asymptomatic volunteers (31 males and 34 females, age range 22–59 years, mean 37.6) were included in this study. All subjects signed an approved informed consent form (Internal Review Board Approval No. 00042801). Exclusion criteria included previous spinal surgery, history of low back pain, age > 60 years, obesity, and claustrophobia or other contraindication to MR imaging.

MR imaging was performed with T2-weighted sequences using a 1.5 T MR imaging scanner (Signa, General Electric, Milwaukee, WI). The imaging protocol included a clinical T2-weighted sequence acquired in the sagittal plane (echo time 125 ms, repetition time > 4300 ms, FOV 300 mm, matrix 512 × 512, spatial resolution: in-plane pixel size 0.59 mm, slice thickness 3 mm). The images were stored in Digital Imaging and Communication in Medicine (DICOM) format, then transferred to personal computers. Image-based analyses were carried out on the mid-sagittal image of each intervertebral disc. Additionally, since this could only be seen after the imaging was completed, discs with defects at the adjacent endplates or Schmorl’s node were excluded from this study. The degenerative stage of each lumbar intervertebral disc was graded by 3 spine surgeons using Pfirrmann grading [7]. A total of 288 lumbar intervertebral discs with clearly identifiable nucleus pulposus boundary (Grade I: *n* = 47, Grade II: *n* = 173, Grade III: *n* = 68) were included in this study. There were no instances of significant bone marrow edema or subchondral sclerosis at the adjacent vertebral bodies of the studied intervertebral discs.

**Fig. 1:**
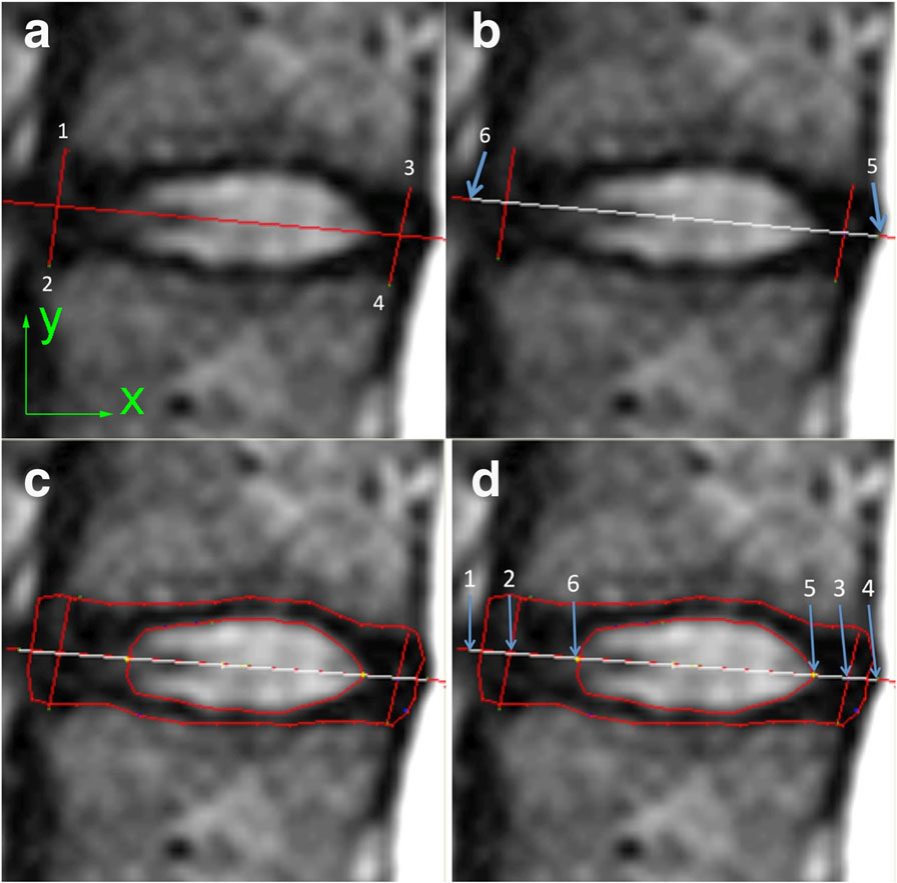
Workflow to obtain the nucleus pulposus edge coordinates. **a** Determine the longitudinal axis of the intervertebral disc by clicking on the corners of adjacent vertebrae in a given sequence. **b** Click on the margins of the intervertebral disc on the longitudinal axis. **c** Mark the contour of the nucleus pulposus and the intervertebral disc. **d** Output of 6 sets of coordinates along the longitudinal axis

A custom routine written in Microsoft Visual C++ 2005 (Microsoft Foundation Class) was used to determine the longitudinal axis of each intervertebral disc, to define the boundary of each nucleus pulposus on the mid-sagittal images, and to locate the coordinates of the geometric and weighted centers of the nucleus pulposus. The weighted center is a hypothetical concept meant to demonstrate the homogeneity within the boundary of the nucleus pulposus by calculating the distance between this weighted center and the geometric center. When the nucleus pulposus is homogeneous (as in a Grade 1 disc) both centers tend to coincide as the pixel intensity is similar. The greater the degeneration grade, the larger the distance between these two locations, given that the pixel intensity varies, causing the weighted center to move towards the area with higher intensity. A step-by-step sequence of the on-screen steps to carry out the image processing analysis is shown in Fig. 1. Analysis of MR image data was performed by two experienced observers (both spine surgeons). The coordinates of the weighted center (*X*_*wc*_, *Y*_*wc*_) were determined according to these equations: *X*_*wc*_ = (Σ*X*_*i*_ *S*_*i*_)/Σ*S*_*i*_; *Y*_*wc*_ = (Σ*Y*_*i*_ *S*_*i*_)/Σ*S*_*i*_; where *X*_*i*_ and *Y*_*i*_ are the coordinates of each pixel and *S*_*i*_ the signal intensity of each pixel [9]. To compare the relationship between these two centers in different anatomic levels, a new local coordinate system for each disc level was set at the geometric center of the intervertebral disc, with the longitudinal axis of the corresponding disc as the new X axis. At the geometric center, a line perpendicular to the longitudinal axis of the disc defined the new Y axis. The locations of geometric and weighted centers of the nucleus pulposus in the new local coordinate system were analyzed. The distances between the weighted center and the geometric center on the longitudinal axis and the perpendicular axis of the intervertebral disc were calculated.

### Statistical analyses

Distances between the geometric and weighted centers at each intervertebral disc were sought with ANOVA. A Fisher’s protected least significant difference (PLSD) test was used to compare between two groups of different Pfirrmann grades. Statistical significance was set at *p* < 0.05. Inter- and intra-class correlation coefficients were computed for all 288 measurements carried out by both observers (SPSS v. 22.0; IBM, Armonk, NY). This rating was done for both the Pfirrmann grading as well as the nucleus pulposus homogeneity assessments.

## Results

For the Pfirrmann grading, ranges for intra- and inter-observer correlation coefficients were [0.794–0.885] and [0.670–0.749], respectively. When it comes to measurements determining geometric and weighted centers, the ranges for intra- and inter-observer correlation coefficients were [0.853–1.000] and [0.857–1.000], respectively.

**Fig. 2:**
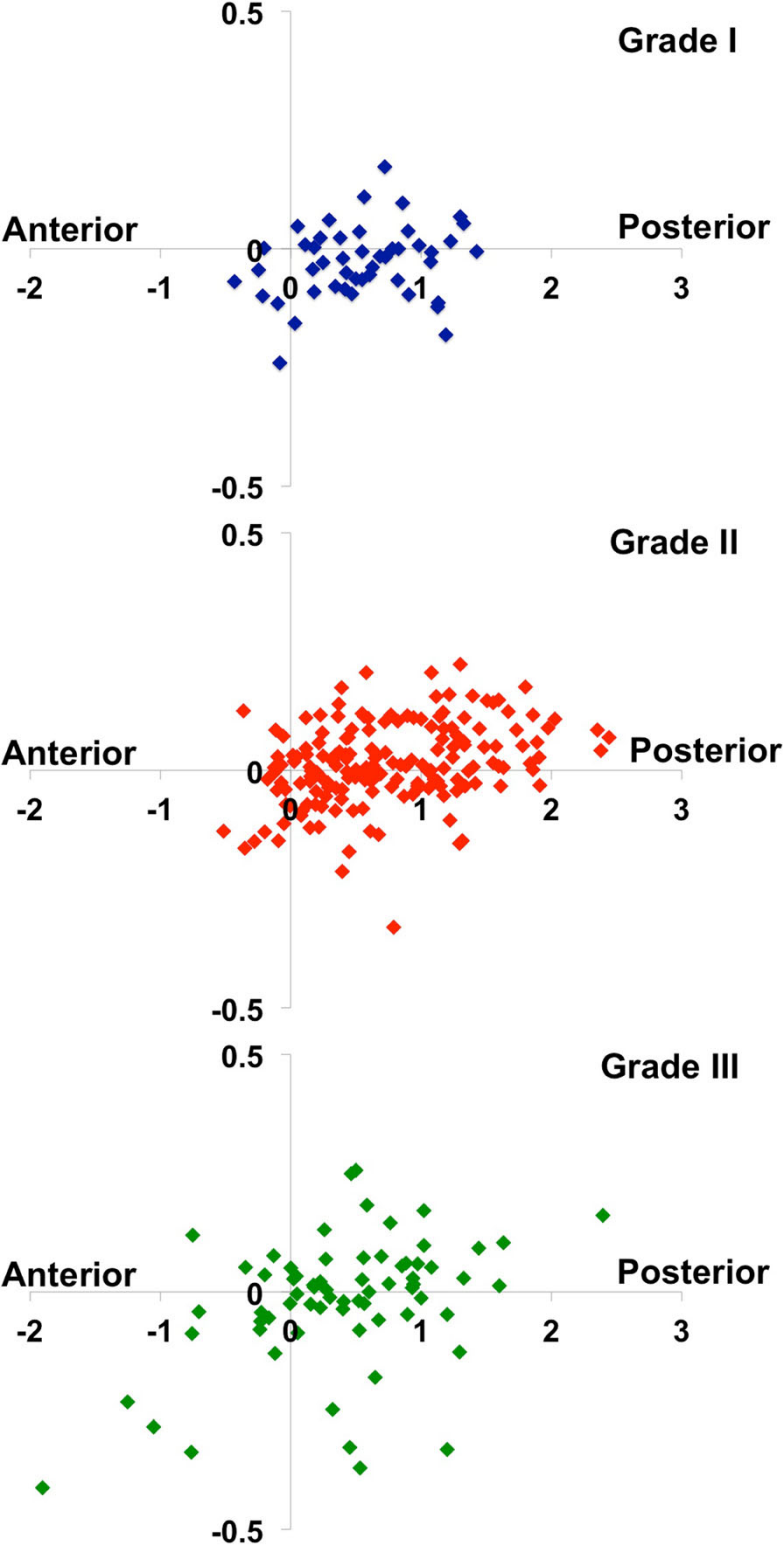
Distribution of center coordinates on the sagittal images. The center weighted by signal intensity located posteriorly to the geometric center of the nucleus pulposus in 87.2% of Pfirrmann Grade I discs, 89.0% of Grade II discs and 76.5% of Grade III discs. The scale of the perpendicular axes was exaggerated in order to show the distribution in the superior-inferior direction in all three plots

On the longitudinal axis (anterior-posterior direction), the weighted center located posterior to the geometric center in 85.8% of the nuclei whose boundary was clearly identifiable. This finding indicated that the signal intensity was lower at the anterior portion than at the posterior portion in the vast majority of discs (Fig. 2). In intervertebral discs of different Pfirrmann grading, this phenomenon was found in 87.2% of Grade I discs, 89.0% of Grade II discs, and 76.5% of Grade III discs. This phenomenon was also observed in different anatomic levels. Although the prevalence was higher in upper lumbar discs, this phenomenon was still dominant in early degenerative stages of lower lumbar discs (L1/2 91.7%, L2/3 91.5%, L3/4 83.9%, L4/5 86.7%, L5/S1 72.3%).

On the perpendicular axis, the weighted center did not locate predominantly either superior (50.3%) or inferior (49.7%) to the geometric center in this study. However, the weighted centers were more frequently superior to the geometric centers in the upper lumbar intervertebral discs (L1/2 and L2/3 discs, 59.7%) while more inferiorly located in the lower lumbar intervertebral discs (L4/5 and L5/S1, 60.1%).

**Fig. 3:**
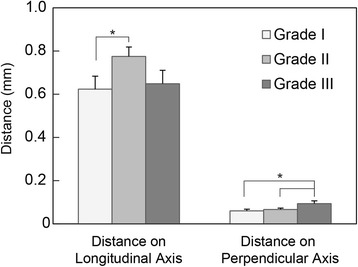
Distances between the weighted center and the geometric center of nucleus pulposus by axis and Pfirrmann grade. The distance on the longitudinal axis in Grade II discs was significantly larger than that in Grade I discs (*p* = 0.035). The distance on the axis perpendicular to the longitudinal axis in Grade III discs was significantly larger than that in Grade I and Grade II discs (*P* < 0.01)

**Fig. 4:**
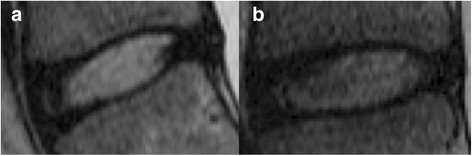
Specific Cases **a** An example of Pfirrmann Grade I disc. The coordinate of the weighted center related to the geometric center was (0.093, −0.038). **b** An example of Pfirrmann Grade II disc. The coordinate of the weighted center related to the geometric center was (1.923, 0.062)

The distances between the weighted center and the geometric center of nucleus pulposus on the longitudinal and perpendicular axes in different Pfirrmann grades are shown in Fig. 3. The average distances (mean ± SD) between these two centers on the longitudinal axis were 0.60 ± 0.06 mm in Grade I discs, 0.78 ± 0.04 mm in Grade II discs, and 0.65 ± 0.06 mm in Grade III discs. The average distances on the axis perpendicular to the longitudinal axis were 0.06 ± 0.01 mm in Grade I discs, 0.07 ± 0.01 mm in Grade II discs, and 0.10 ± 0.01 mm in Grade III discs. The distance on the longitudinal axis in Grade II discs was significantly larger than that in Grade I discs (*p* = 0.035). Representative images demonstrating differences between Grade I and Grade II discs are shown in Fig. 4. The distance on the axis perpendicular to the longitudinal axis in Grade III discs was significantly larger than that in Grade I and Grade II discs (*P* < 0.01).

## Discussion

The distance between the center weighted by signal intensity of T2-weighted MR images and the geometric center of the nucleus pulposus has been used to predict the progression of intervertebral disc degeneration [9]. However, a previous study showed this distance was not sensitive to the variations that occurred with idiopathic scoliosis and spondylolisthesis. In the current study, the same concept was adopted to check its feasibility on determining the changes of signal homogeneity and the progress of degenerative process in the nucleus pulposus through advancing stages of intervertebral disc degeneration. Theoretically, the weighted center and the geometric center should be close to each other in a homogeneous nucleus pulposus. Asymmetric distribution of signal intensity along any given axis on both sides of the geometric center would make the weighted center move away from the portion with lower signal intensity. As the results show in the current study, the relationship between these two centers has provided important information inside the nucleus pulposus at different stages of intervertebral disc degeneration.

The results of the current study showed the weighted center located posterior to the geometric center in the vast majority of nuclei pulposus of Pfirrmann Grade I, II and III discs. This phenomenon remained dominant even in different anatomic levels. This finding reflected the intervertebral discs at early degenerative stages generally had a lower overall signal intensity at the anterior portion of the nucleus pulposus. Although it has never been clearly mentioned in the literature, figures of previous disc diffusion studies have shown enhancement by contrast medium was lower at the anterior corner of the nucleus pulposus [10, 11]. A recent study using quantitative T2* MRI also showed anterior predominant degeneration during initial stages of intervertebral disc degeneration [12]. Differential tissue properties in different regions of the nucleus pulposus may be an explanation for this finding.

A central band of greater fiber content has been shown to cross the equator of the intervertebral disc, producing a band of lower signal intensity in MR imaging [13, 14]. This band of organized fibrous tissue in the equator of the intervertebral disc appeared to indent the amorphous portion of the nucleus pulposus [13] and to develop progressively from the periphery of the nucleus pulposus toward the center [14]. The fibrous portion may be located at anterior or posterior portion of the nucleus pulposus, but the progression after its appearance is still not clear.

As the result of the current study showing that distance on longitudinal axis between the weighted center and the geometric center of the nucleus pulposus in Pfirrmann Grade II discs was larger than in Grade I discs, it suggested the progressive decrease of T2-weighted MR signal intensity was more predominant at the anterior portion of the nucleus pulposus during the early progression of intervertebral disc degeneration. While the degeneration further progressed, T2-weighted MR signal intensity at the posterior portion of the nucleus pulposus decreased which brought the weighted center and geometric center of the nucleus pulposus closer in the Pfirrmann Grade III discs.

**Fig. 5:**
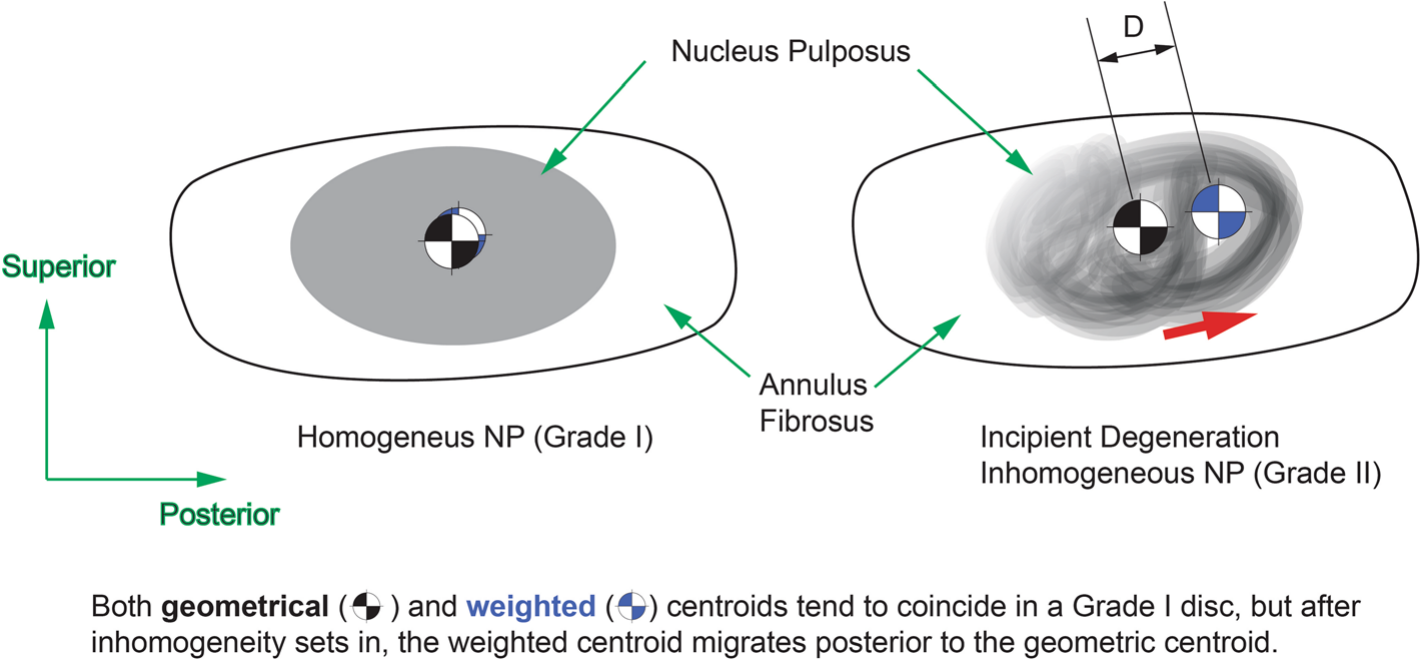
Nucleus pulposus MR signal intensity. A clear pattern emerged with increasing disc degeneration grades with both centroids located further apart from each other

Although the distance on the longitudinal axis between the weighted center and the geometric center was different between Pfirrmann grades I and II discs, the distance on the perpendicular axis was similar in these two groups. As the degeneration progressed to Pfirrmann Grade III, the distance between two centers increased on the perpendicular axis. The distance between two centers through advancing stages of degeneration demonstrated the decrease of T2-weighted MR signal intensity progressing along the longitudinal axis initially and then to perpendicular direction at the later stage. This pattern of signal intensity changes provided an insight on possible progress of tissue property during early stages of intervertebral disc degeneration (Fig. 5).

The strength of the current study is using conventional MR images to evaluate the homogeneity and the progressive changes of T2-weighted MR signal intensity in the nucleus pulposus. This method needs no additional scanning time, administration of contrast medium, or other specific apparatus. However, this study is not without its own limitations. The sample size of this study was still small and a larger scale of study would be needed. In addition, the intervertebral discs analyzed in this work were not subjected to histological, biochemical studies, or the other MR image techniques such as T1-rho and T2-mapping, as it were acquitted in vivo. This was a cross-sectional study using an established database that could only reflect the phenomenon of one time point. A longitudinal study is needed to demonstrate the applicability of this method to quantify or to predict the progression of intervertebral disc degeneration.

This retrospective study was performed based on an existing database of asymptomatic subjects. The relationship between the geometric and weighted centers could not correlate to severity of clinical symptoms. However, it may be used to monitor the progress of degenerative changes in longitudinal studies, to detect early degenerative changes in the intervertebral discs adjacent to spinal fusion, to evaluate animal models after induced degeneration and after treatments.

## Conclusion

Evaluation of the distance between nucleus pulposus weighted and geometric centers may be correlated with Pfirrmann grade. Therefore, it may be used as a complementary quantitative biomarker of intervertebral disc degeneration and help understand the different steps of intervertebral disc degeneration. The distance between two centers through advancing stages of intervertebral disc degeneration demonstrated the decrease of signal intensity progressing along the longitudinal axis initially and then to perpendicular direction at the later stage. These findings could provide an insight of initiation and subsequent progression of degenerative changes in the intervertebral discs.

## Abbreviations

AF: Annulus fibrosus
IVD: Intervertebral disc
MR: Magnetic resonance
NP: Nucleus pulposus
